# Evaluation of Microvascular Structure Changes after Conbercept Treatment on Macular Edema Secondary to Retinal Vein Occlusion

**DOI:** 10.1155/2020/9046781

**Published:** 2020-06-19

**Authors:** Wenqi Song, Wanzhen Jiao, Fengjiao Li, Aihua Ma, Bojun Zhao

**Affiliations:** ^1^Shandong University of Traditional Chinese Medicine, Jinan, China; ^2^Department of Ophthalmology, Shandong Provincial Hospital Affiliated to Shandong First Medical University, Jinan, China; ^3^Department of Pediatric, Shandong Provincial Hospital affiliated to Shandong first Medical University, Jinan, China

## Abstract

**Aims:**

To confirm the therapeutic efficacy of conbercept for the treatment of macular edema (ME) secondary to retinal vein occlusion (RVO) by using optical coherence tomography angiography (OCTA) and to find out the differences in therapeutic efficacy between ischemic and nonischemic retinal vein occlusion (iRVO or non-iRVO) after conbercept treatment.

**Methods:**

In this prospective, randomized, and comparative study, 60 unilateral eyes suffered from RVO combined with macular edema were included and fellow eye as controls. After an initial intravitreal injection of conbercept (IVIC), a pro re nata (PRN) strategy was adopted, and the follow-up time was 6 months. The foveal avascular zone (FAZ), vascular density of superficial capillary plexus (SCP), and vascular density of deep retinal capillary plexus (DCP), nonperfused areas (NPAs) were evaluated with OCTA on baseline and after treatment.

**Results:**

The mean intravitreal injection number was 2.9 ± 0.89 times during six months in iRVO patients and 2.1 ± 0.86 times in non-iRVO patients, with statistically significant difference (*p* < 0.05). On baseline, central macular thickness (CMT) and FAZ were significantly thickened and enlarged compared to those of healthy fellow eyes; the vascular density of SCP and DCP were significantly decreased, and the differences were statistically significant (*p* < 0.05). Compared to baseline, after treatment, the best-corrected visual acuity (BCVA) was improved in either iRVO or non-iRVO (−0.601 ± 0.387, −0.241 ± 0.341 logMAR, *p* < 0.05). In iRVO, the improvement was more substantial than that of the non-iRVO group. FAZ in the non-iRVO group had significantly decreased compared to that in iRVO group (−0.044 ± 0.040 versus 0.014 ± 0.043 mm^2^, *p* < 0.05). CMT, the vascular density of SCP, and DCP had no significant difference.

**Conclusions:**

The changes of microvascular structure can be quantitatively evaluated by using OCTA for the patients with RVO. Conbercept had a significant effect on treatment of RVO with macular edema. A more profound effect was achieved in the iRVO group on visual improvement and FAZ reduction in the non-iRVO group after conbercept treatment.

## 1. Introduction

Retinal vein occlusion (RVO) is the second most common cause of retinal vascular disease and causes haemorrhage and macular edema, leading to significant loss of vision after diabetic retinopathy [1]. The main treatment modalities include laser therapy, intravitreal injections of steroids, and vascular endothelial growth factor (VEGF) inhibitors. Compared to grid laser and steroids, patients treated with VEGF inhibitors demonstrated excellent visual gain and anatomic improvement; therefore in recent years, the anti-VEGF therapy has become more prevalent in the treatment of visual loss associated with macular edema (ME) [2]. The beneficial effect of conbercept is mainly due to its anti-VEGF action which is able to decrease vascular permeability in retina [3]. Recently, conbercept has been used in the treatment of RVO and achieved satisfactory results [4, 5]. However, the side effects of blocking VEGF remain a concern; one of which is whether anti-VEGF therapy impairs retinal blood circulation and facilitates retinal vascular occlusion in eyes with diabetic retinopathy and RVO [6, 7].

In recent years, the development of noninvasive optical coherence tomography angiography (OCTA) enabled mapping of several retinal and choroidal vascular layers [8]. Moreover, OCTA has been used to measure the area of the foveal avascular zone (FAZ), vascular density, and nonperfused areas (NPAs) [9]. Furthermore, OCTA can visualize NPAs better than that of fluorescein angiography (FA) in RVO [10]. The manifestation on retinal vein occlusion under OCTA examination has been recently reported [11, 12].

This study was designed to evaluate the difference between affected eyes and fellow eyes with RVO patients with quantitative OCTA parameters. Another aim of this study was to compare the difference between eyes with ischemic and non-ischemic RVO with quantitative OCTA parameters after intravitreal injection of conbercept (IVIC) and to observe the change of blood circulation after anti-VEGF treatment.

## 2. Materials and Methods

This study was a prospective and comparative study. Patients were recruited from January to October, 2018, at Shandong Provincial Hospital. The study was approved by the Medical Ethics Committee of Shandong Provincial Hospital affiliated to Shandong First Medical University. All participants provided signed written informed consent.

### 2.1. Patients

Inclusion criteria were as follows: (1) age ≥ 18 years and (2) patients diagnosed with RVO after fundus examination, FFA, OCT, OCTA and other related examinations. Exclusion criteria were as follows: (1) the patients declined to join the study; (2) vitrectomy, retinal laser photocoagulation, glucocorticoid, and anti-VEGF therapy were performed before participating in this study; (3) other retinal vascular diseases or eye diseases affecting vision; (4) severe systemic disease; (5) female patients during lactation or pregnancy; and (6) those who were unable to cooperate with the study or withdraw during the study.

### 2.2. Patient Assignment

At baseline, a fluorescein angiography (SPECTRALIS, version 1.9.10.0, Heidelberg Engineering, Heidelberg, Germany) was carried out for all the patients to detect the presence of peripheral and macular ischemic areas. The RVO subtypes (ischemic and non-ischemic) were determined by two masked retinal specialists. Branch retinal vein occlusions (BRVO) with a nonperfusion area larger than 5 disc diameters and central retinal vein occlusions (CRVO) with a non-perfusion area larger than 10 disc areas on FA examination were defined as an ischemic type [7]. Patients were treated with one injection of conbercept followed by PRN strategy with monthly assessments. The interval of injections was no less than once every 4 weeks. Eyes were retreated if either of the following conditions occurred during follow-up: (i) >2 lines of vision loss in Snellen acuity or (ii) >50 *μ*m in CMT compared to that of previous measurement; and (iii) persistent or increased intraretinal and/or subretinal fluid.

The BCVA and central macular thickness (CMT) (OCT, Optovue, USA) were measured at baseline and 6 months after treatment, respectively. OCTA (Optovue, RTVue-XR Avanti, USA) was done for all participants using the split spectrum amplitude decorrelation angiography algorithm. For each eye, images were taken with the size 6 × 6 mm in macular area. The images of FAZ area (by automated segmentation from internal limiting membrane (ILM) to 10 *μ*m below the outer plexiform layer (OPL0), SCP (by automated segmentation from ILM to external boundary of ganglion cell layer), and DCP (by automated segmentation from inner plexiform layer to OPL) were analyzed with the software of AngioAnalytics. Vascular density was calculated as a percentage of the area occupied by vessels in selecting area. Two masked retinal specialists determined the NPAs by the AngioAnalytics software.

### 2.3. The Intravitreal Injection Procedure

The injections were performed in the operating theater with a sharp 29-gauge needle. The needle was inserted into the eye through the pars plana (3.5-4 mm posterior from the limbus), 0.05 ml of solution containing 0.5 mg of conbercept was injected.

### 2.4. Outcome Measurement

The BCVA, CMT, FAZ, SCP, DCP, and NPAS of affected eyes and healthy fellow eyes were measured before and after conbercept treatment.

### 2.5. Statistical Analyses

All statistical analyses were carried out by using SPSS statistical software version 21.0 (SPSS, Inc., Chicago, IL. USA). Data were presented as mean ± standard error. Snellen VAs were converted to logMAR units for analysis. Changes in each parameter before and after treatment were compared using paired sample *t*-tests. Differences between treatment groups were compared using independent sample *t*-tests. A *p* value of <0.05 was considered statistically significant.

## 3. Results

### 3.1. Characteristics of Patients

60 unilateral eyes suffered from unilateral RVO with macular edema were used in the study. Healthy fellow eyes were used as controls. 32 eyes with ischemic type (mean age 55.57 ± 15.43 years) were included in group 1; 28 patients with nonischemic type (mean age 58.19 ± 8.001 years) were included in group 2. Baseline characteristics of patients in iRVO and non-iRVO subgroups were comparable (Table 1).

CMT in non-iRVO eyes and iRVO eyes were significantly thickened compared to that in healthy controls: 494.4 ± 196.88 *μ*m versus 229.8 ± 13.47 *μ*m; 425.5 ± 109.35 *μ*m versus 241.9 ± 12.54 *μ*m, respectively. FAZ in both non-iRVO eyes and in iRVO eyes were significantly enlarged compared to that of healthy fellow eyes on baseline: 0.35 ± 0.02 mm^2^ versus 0.29 ± 0.06 mm^2^; 0.38 ± 0.04 mm^2^ versus 0.30 ± 0.04 mm^2^, respectively. The vascular density of SCP and DCP in non-iRVO eyes and iRVO eyes was also significantly decreased compared to that in healthy controls: 42.89 ± 3.22% versus 48.6 ± 3.91%; 41.5 ± 3.92% versus 48.6 ± 2.57%and 40.7 ± 2.32% versus 49.5 ± 2.36%; 38.8 ± 2.83% versus 49.7 ± 3.46%, respectively, and the differences were statistically significant (*p* < 0.05) (Table 2).

**Figure 1:**
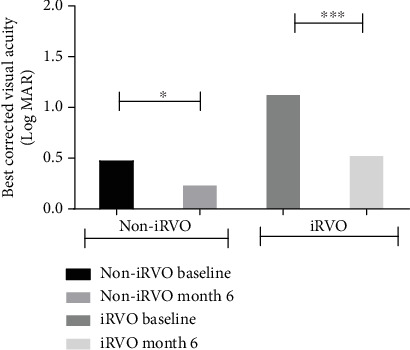
BCVA was improved in both non-iRVO and iRVO groups after conbercept therapy. ^∗^*p* < 0.05, ^∗∗∗^*p* < 0.001.

**Figure 2:**
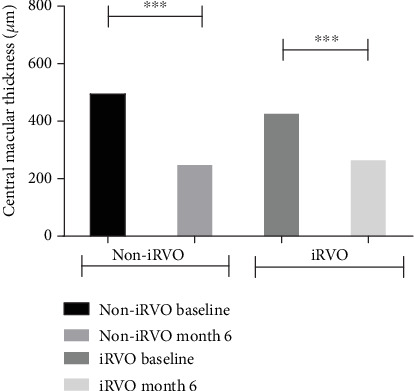
CMT was decreased in both non-iRVO and iRVO after 6-month therapy. ^∗∗∗^*p* < 0.001.

**Figure 3:**
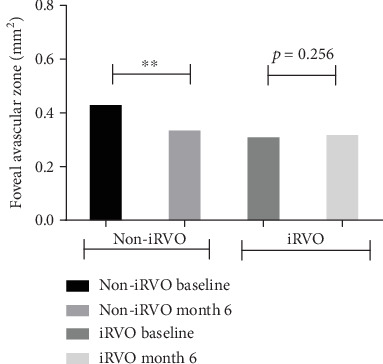
FAZ was decreased in non-iRVO, and no significant change in iRVO after 6-month therapy. ^∗∗^*p* < 0.01.

**Figure 4:**
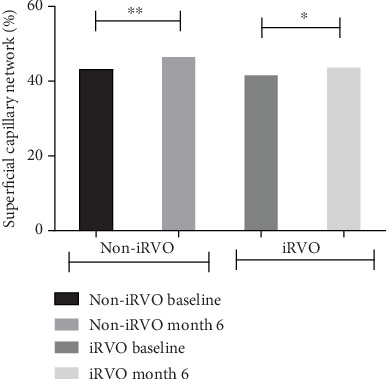
SCP was increased in both non-iRVO and iRVO after 6-month therapy. ^∗^*p* < 0.05, ^∗∗^*p* < 0.01.

**Figure 5:**
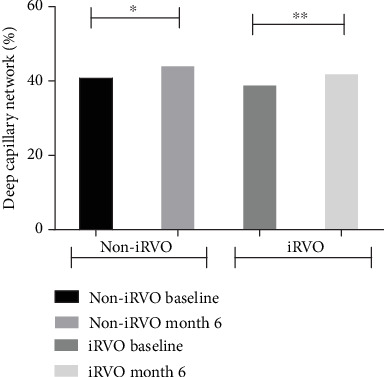
DCP was increased in both non-iRVO and iRVO after 6-month therapy. ^∗^*p* < 0.05, ^∗∗^*p* < 0.01.

**Figure 6:**
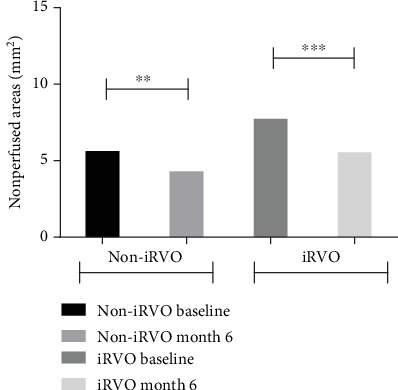
NPAs were decreased in both non-iRVO and iRVO after 6-month therapy. ^∗∗^*p* < 0.01, ^∗∗∗^*p* < 0.001.

**Figure 7:**
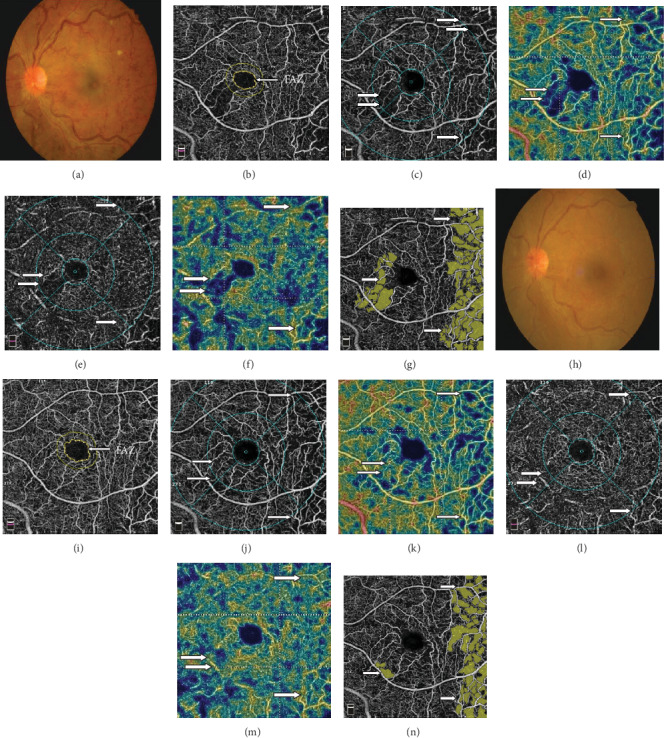
Fundus photo and OCTA of a 53-year-old male with iRVO in the left eye before (a–g) and 6 months after treatment (h–n). Fundus photo (a, h) showed marked resolution of retinal hemorrhage after treatment. OCTA images showed that the FAZ (b, i) encircled by the yellow line was enlarged after treatment. The capillary perfusion (white arrow) was increased after treatment in both the SCP (j, k) and DCP (l, m) layers compared to that of SCP (c, d) and DCP (e, f) before treatment. The areas of NPAs (white arrow) decreased after treatment (n) compared to that before treatment (g).

Compared to baseline, at the end of follow-up, in the iRVO group, BCVA was significantly improved (1.12 ± 0.48 versus 0.52 ± 0.47 logMAR, *p* < 0.05) (Figure 1) and CMT was significantly decreased (425.50 ± 109.35 versus 265.00 ± 44.79 *μ*m, *p* < 0.01) (Figure 2). FAZ had not changed significantly (0.375 ± 0.044 versus 0.389 ± 0.046 mm^2^, *p* > 0.05) (Figure 3). Both vascular density SCP and DCP were increased significantly (SCP: 41.53 ± 3.92 versus 43.64 ± 5.57%, *p* < 0.05; DCP: 38.78 ± 2.825 versus 41.81 ± 4.260%, *p* < 0.01 Figures 4 and 5 ). NPAS were significantly decreased (6.963 ± 4.379 versus 5.539 ± 3.444 mm^2^, *p* < 0.01) (Figure 6). In the non-iRVO group, BCVA were significantly improved (0.47 ± 0.32 versus 0.23 ± 0.17 logMAR, *p* < 0.01) (Figure 1) and CMT were significantly decreased (494.43 ± 196.88 versus 247.07 ± 48.60 *μ*m, *p* < 0.01) (Figure 2). In contrast to iRVO, FAZ was significantly reduced (0.345 ± 0.024 mm^2^ versus 0.301 ± 0.039 mm^2^, *p* < 0.01) (Figure 3). The vascular density of SCP and DCP was significantly increased (SCP: 42.89 ± 3.22 versus 46.07 ± 3.30%, *p* < 0.01; DCP: 40.72 ± 2.323 versus 44.32 ± 5.387%, *p* < 0.05, Figures 4 and 5). NPAS were significantly decreased (5.635 ± 2.668 versus 4.289 ± 2.647 mm^2^, *p* < 0.01) (Figure 6). Representative images of the changes in fundus photo, FAZ, SCP, DCP, and NPAs before and after treatment are shown in Figure 7.

Before and after treatment, the changes of various parameters between the non-iRVO group and the iRVO group were compared. The improvement of BCVA (−0.241 ± 0.341 versus −0.601 ± 0.387 logMAR, *p* < 0.05) was more substantial in the iRVO group than that in the non-iRVO group. The reduction of FAZ (−0.044 ± 0.030 versus 0.014 ± 0.043 mm^2^, *p* < 0.05) was more substantial in the non-iRVO group than that in the iRVO group, and the difference was statistically significant. The changes of CMT (−247.36 ± 193.53 versus −160.50 ± 110.31 *μ*m, *p* > 0.05) and the vascular density of SCP (3.407 ± 3.268 versus 2.050 ± 3.321%, *p* > 0.05) and DCP (3.600 ± 5.598 versus 3.031 ± 3.055%, *p* > 0.05) showed no significant difference between the two groups (Table 3).

The mean intravitreal injection was 2.9 ± 0.89 times in iRVO patients and 2.1 ± 0.86 times in non-iRVO patients, with statistically significant difference (*p* < 0.05).

## 4. Discussion

RVO is a common retinal vascular disease. Occlusion causes ischemia and hypoxia of the retina, which can lead to a rise in the level of VEGF [13]. VEGF can induce a breakdown in the blood–retinal barrier and increase vascular permeability, which result in ME [14]. A number of studies have reported that the concentration of VEGF is elevated in the vitreous of RVO patients, indicating that VEGF is an important factor in the severity of ME [15].

Conbercept is a novel human-derived anti-VEGF recombinant fusion protein developed in China (Chengdu Kanghong Biotech Co., Ltd., Sichuan, China), which can inhibit the activity of VEGF, and effectively penetrate the whole retinal layer. Its molecular weight is 143 kDa. It contains extracellular domain-2 of VEGFR-1 and extracellular domains-3 and-4 of VEGFR-2. The structure is similar to aflibercept, but they differ in that conbercept contains a fourth VEGFR-2 binding domain, which may enhance the association rate of VEGF and prolong its half-life in the vitreous. Compared to ranibizumab and bevacizumab, the most notable characteristic of conbercept is that it binds not only to VEGF-A but also to VEGF-B and placental growth factor with high affinity [16]. Furthermore, the preclinical studies have shown that conbercept has advantages over ranibizumab and bevacizumab in that it has a longer half-life and a stronger binding affinity to VEGF-A [17]. Conbercept has been approved to treat neovascular age macular degeneration by the China Food and Drug Administration in December 2013. The efficacy of conbercept in the treatment of polypoidal choroidal vasculopathy has been reported [18]. We have also reported that the conbercept is effective in the treatment of ME secondary to RVO [4]. The aim of this prospective study was to investigate the clinical efficacy of conbercept for treatment of non-iRVO and iRVO.

OCTA has recently been introduced into clinical practice which permits the visualization of the different retinal vascular layers and the monitoring of the vascular changes in RVO [19]. OCTA as a noninvasive method providing microvascular assessment using blood cell movement as natural contrast [20].

In this study, we quantitatively measured the vascular density and FAZ using OCTA in patients with RVO and compared them to unaffected fellow eyes. Our result demonstrated that all of the blood flow density decreased and FAZ enlarged in the affected eyes. It can be partially explained by the presence of retinal vein occlusion which increases the intravascular pressure and hydrostatic pressure leading to decrease in flow. The cells in FAZ and surrounding area are mainly nourished by choroid. Under physiological conditions, choroid circulation can meet the metabolic requirements of FAZ and surrounding areas. However, when retinal blood vessels are damaged, they cannot get enough oxygen from the choroid, leading to rupture of arch ring and enlargement of FAZ. Similar results were reported in previous studies. Suzuki et al. found that in 16 patients with monocular RVO, the area of FAZ in the retinal deep capillary layer of RVO eyes was larger than that in unaffected fellow eyes [21]. Khodabandeh et al. reported 31 patients with CRVO and 20 cases as healthy controls, the results exhibited that the decreased in vascular density in both SCP and DCP with affected eyes of RVO [22].

Capillary narrowing or occlusion occurring in ischemic retina leads to tissue hypoxia with subsequent increase in VEGF levels in RVO patients. In this study, intraocular injections of conbercept were performed on patients with RVO secondary ME. Each parameter of the patients at baseline and at month 6 of follow-up time in patients treated with conbercept was recorded. The first finding was that the FAZ area in eyes with non-iRVO decreased 6 months after anti-VEGF therapy. However, the FAZ area had no significant differences in eyes with iRVO, which may be due to the ischemia which had caused nonreversible damage of retinal capillary in iRVO patients. Deng et al. [23] reported that the FAZ area was statistically unchanged during the course of 1 month of conbercept therapy in RVO patients. One of the reasons may be due to the limited sample size and short observation period. Our second finding was that the vascular density in the eyes with RVO was improved 6 months after anti-VEGF therapy, accompanied by decreasing NPAs in both non-iRVO and iRVO. The therapy of anti-VEGF appears to play a major role in vascular remodeling and development of collateral vessels, which further decreases vascular permeability and macular edema and improves the retinal vascular flow. Campochiaro et al. [14] reported that blockade of VEGF not only has prevented deterioration in of NPAs but also might have improved the retinal perfusion status. Our current results corroborate this hypothesis.

Various parameters between the iRVO group and the non-iRVO group on baseline and after treatment were compared. It has been reported that VEGF concentration in aqueous humor and vitreous is significantly higher in iRVO than that in non-iRVO [24]. Thus, the intravitreal injection numbers in iRVO was significantly higher than that in the non-iRVO. The increased intravenous hydrostatic pressure occurring following RVO leads to a decrease in the retinal capillary perfusion, which further leads to capillary nonperfusion. The affected area of the retina is more extensive in iRVO compared to that in non-iRVO. Due to the poor baseline vision, the improvement of BCVA was greater in the iRVO group than that of the non-iRVO group after treatment. Both the ischemic and the nonischemic groups had similar therapeutic effects in CMT, SCP, and DCP after conbercept treatment in our observation. Randomized multicentered clinical trials with larger samples and longer follow-up periods are necessary to further confirm this observation.

Taken together, conbercept has a significant effect on the treatment of ME secondary RVO for both non-iRVO and iRVO. OCTA can accurately evaluate the changes of microvascular structure in patients with RVO and therefore can be used as a valuable imaging tool for the follow-up both in non-iRVO and iRVO patients.

## Figures and Tables

**Table 1:** Baseline characteristics of subjects.

	Non-iRVO eyes	iRVO eyes	*p*
*N* (eyes)	28	32	—
Gender (male : female)	16 : 12	14 : 18	0.961
Age (years)	55.57 ± 15.43	58.19 ± 8.001	0.557
IOP, mmHg (mean ± SD)	13.64 ± 1.216	14.19 ± 2.040	0.391
Hypertension (yes : no)	18 : 10	24 : 8	0.523
BRVO : CRVO	16 : 12	14 : 18	0.961

*N*: number of eyes; non-iRVO: nonischemic retinal vein occlusion; iRVO: ischemic retinal vein occlusion; BRVO: branch retinal vein occlusion; CRVO: central retinal vein occlusion.

**Table 2:** The parameters of the affected eyes compared to unaffected fellow eyes of RVO patients.

	Non-iRVO eyes	Follow eyes	*p*	iRVO eyes	Follow eyes	*p*
CMT	494.43 ± 196.88	229.79 ± 13.47	*p* < 0.01	425.50 ± 109.35	241.88 ± 12.54	*p* < 0.01
FAZ	0.345 ± 0.024	0.290 ± 0.063	*p* < 0.01	0.375 ± 0.044	0.295 ± 0.039	*p* < 0.01
SCP	42.89 ± 3.22	48.52 ± 3.907	*p* < 0.01	41.53 ± 3.92	48.55 ± 2.565	*p* < 0.01
DCP	40.72 ± 2.323	49.47 ± 2.359	*p* < 0.01	38.78 ± 2.825	49.72 ± 3.460	*p* < 0.01

All data were presented as mean ± SD. CMT: central macular thickness; FAZ; foveal avascular zone; SCP; superficial capillary plexus; DCP: deep capillary plexus.

**Table 3:** The changes in parameters of non-iRVO and iRVO eyes before and after the treatment.

	Non-iRVO eyes	iRVO eyes	*t*	*p*
BCVA	−0.241 ± 0.341	−0.601 ± 0.387	2.698	0.012
CMT	−247.36 ± 193.53	−160.50 ± 110.31	-1.482	0.154
FAZ	−0.044 ± 0.030	0.014 ± 0.043	-4.907	<0.01
SCP	3.407 ± 3.268	2.050 ± 3.321	1.125	0.270
DCP	3.600 ± 5.598	3.031 ± 3.055	0.352	0.728

## Data Availability

The datasets generated and analyzed during the current study are not publicly available but all are kept at Shandong Provincial Hospital affiliated to Shandong first Medical University and available from the corresponding author (Bojun Zhao) on reasonable request.
